# Inhibition of the voltage-gated potassium channel Kv1.5 by hydrogen sulfide attenuates remodeling through S-nitrosylation-mediated signaling

**DOI:** 10.1038/s42003-023-05016-5

**Published:** 2023-06-19

**Authors:** Moza M. Al-Owais, Nishani T. Hettiarachchi, Mark L. Dallas, Jason L. Scragg, Jonathan D. Lippiat, Arun V. Holden, Derek S. Steele, Chris Peers

**Affiliations:** 1 School of Biomedical Sciences, Faculty of Biological Sciences, University of Leeds, Leeds, LS2 9JT UK; 2 Division of Cardiovascular and Diabetes Research, LICAMM, Faculty of Medicine and Health, University of Leeds, Leeds, LS2 9JT UK; 3 Reading School of Pharmacy, University of Reading, Reading, RG6 6UB UK

**Keywords:** Cardiovascular biology, Atrial fibrillation

## Abstract

The voltage-gated K^+^ channel plays a key role in atrial excitability, conducting the ultra-rapid rectifier K^+^ current (I_Kur_) and contributing to the repolarization of the atrial action potential. In this study, we examine its regulation by hydrogen sulfide (H_2_S) in HL-1 cardiomyocytes and in HEK293 cells expressing human Kv1.5. Pacing induced remodeling resulted in shorting action potential duration, enhanced both Kv1.5 channel and H_2_S producing enzymes protein expression in HL-1 cardiomyocytes. H_2_S supplementation reduced these remodeling changes and restored action potential duration through inhibition of Kv1.5 channel. H_2_S also inhibited recombinant hKv1.5, lead to nitric oxide (NO) mediated S-nitrosylation and activated endothelial nitric oxide synthase (eNOS) by increased phosphorylation of Ser1177, prevention of NO formation precluded these effects. Regulation of I_kur_ by H_2_S has important cardiovascular implications and represents a novel and potential therapeutic target.

## Abstract (web summary)

Hydrogen sulfide regulates Kv1.5 channel activity through S-nitrosylation-mediated signaling in HL-1 cardiomyocytes and HEK293 cells expressing human Kv1.5.

## Introduction

The cardiac action potential duration (APD) depends on cycle length, it decreases as heart rate increases as shown in dynamic and static APD restitution curves, which can be quantitatively explained by slow changes in intracellular compartmental ionic concentrations, sarcolemmal pump and exchanger activity, and the activation and inactivation properties of ionic channels^1^. However, APD can also be influenced by long term patterns of cardiac activity^2^, leading to changes in ion channel expression and associated currents^3–5^. Such changes have been demonstrated in vivo, following sustained periods of high frequency activity induced by repetitive stimulation^6^, or following sustained tachycardia^7^ or atrial fibrillation^8^ (AF). Comparable effects have been demonstrated in vitro, for example, in cultured mouse HL-1 atrial myocytes^9^ or rat atrial cells^10^. This epigenetic “remodeling” takes hours or days to develop, and may involve changes in the expression, synthesis, transport, insertion or recycling of channels, their protein subunits and associated regulatory proteins^3–5,11^. However, remodelling can also occur due to downstream changes in the pathways that regulate ion channel function. Importantly, APD shortening can itself promote arrhythmias, e.g. in AF, APD shortening facilitates re-entry and sustains the arrhythmia^12^.

Electrophysiological changes in chronic AF involve downregulation of L-type calcium current (I_Ca,L_), the transient outward current (I_to_) and upregulation of steady-state outward current, the inward rectifier potassium current (I_KI_) and the acetylcholine-activated potassium current (I_KACh_)^3,13^. Computational modelling has shown that these changes in channel regulation (modelled by changes in maximal conductances) reproduce the shortened APDs characteristic of AF and emphasise the important of changes in K^+^-selective channels^4,14,15^. Although different pathophysiological mechanisms contribute to AF^16–19^, one key factor appears to be redox status. AF is clearly associated with increased reactive oxygen and nitrogen species (ROS/RNS), although their source within atrial tissue differs between the acute and chronic forms of AF^20–24^. Also associated with AF is the downregulation of endothelial nitric oxide synthase, (NOS3/eNOS) and reduced nitric oxide (NO) bioavailability^25,26^. There is an emerging view that specific targeting of ROS/RNS production, protection of target proteins, or control of NO bioavailability, may reduce or reverse the transcription and electrophysiological remodelling of AF^20^.

Numerous proteins have been implicated both as redox-sensitive and of central importance in AF^24,27^. In particular, the rapidly activating voltage-gated K^+^ channel Kv1.5, responsible for the ultrarapid delayed rectifier current I_Kur_ that contributes significantly to atrial repolarization^28,29^ and is a potential therapeutic target. Expression of Kv1.5 is largely confined to the atria^30^, and it has been shown that reduction of I_Kur_ prolongs the duration of action potential in remodelled atrial cells^31^. Indeed, a high prevalence of deleterious AF-associated mutations has been identified in KCNA5, the gene encoding Kv1.5, causing both gain- and loss-of channel function in lone AF patients^32,33^. This seems paradoxical, but AF has been proposed to arise from (i) a prolonged effective refractory period, which increases the likelihood of early afterdepolarization arrhythmias, which could arise from loss-of-function Kv1.5 mutants^34^ or (ii) a reduced effective refractory period, increasing the likelihood of re-entrant arrhythmias, which could arise from gain-of-function Kv1.5 mutants^32^. Thus, either excessive or insufficient Kv1.5 activity can lead to AF-associated arrhythmia.

The involvement of the biologically active gas hydrogen sulfide (H_2_S) in modulating cardiovascular functions^35,36^ has been established over the past decade. H_2_S is considered an important signaling molecule and along with NO and carbon monoxide (CO), are designated ‘gasotransmitters’^36,37^. Endogenous H_2_S is a product of distinct, widely distributed enzymes; primarily derived from L-cysteine via the enzymes cystathionine-γ-lyase (CSE) and cystathionine-β-synthase (CBS), but also from 3-mercaptopyruvate sulfurtransferase (3-MPST) along with cysteine aminotransferase (CAT)^38^. Within the cell cytosol or mitochondria, H_2_S can be produced from the intracellular sulphur bound to proteins (sulfane sulfur) in a redox- or pH- dependent manner^39^.

While the modulation of Kv1.5 by NO and CO has been studied previously^40,41^, the effect of H_2_S has not. H_2_S is linked to diverse physiological functions including regulation of blood pressure^42^, and sulfhydryl modulation of smooth muscle K_ATP_ channels^43,44^. Therefore, in the present study, we identified and characterised the modulation of Kv1.5 by H_2_S, in HL-1 cardiomyocytes in which electrical remodelling was induced by high frequency stimulation. We also investigated the underlying signaling pathways, which provide a novel and potentially important target for reversal of the electrical remodeling and the treatment of AF.

## Results

### Interval pacing of HL-1 cardiomyocytes causes electrical and cellular remodeling

**Fig. 1:**
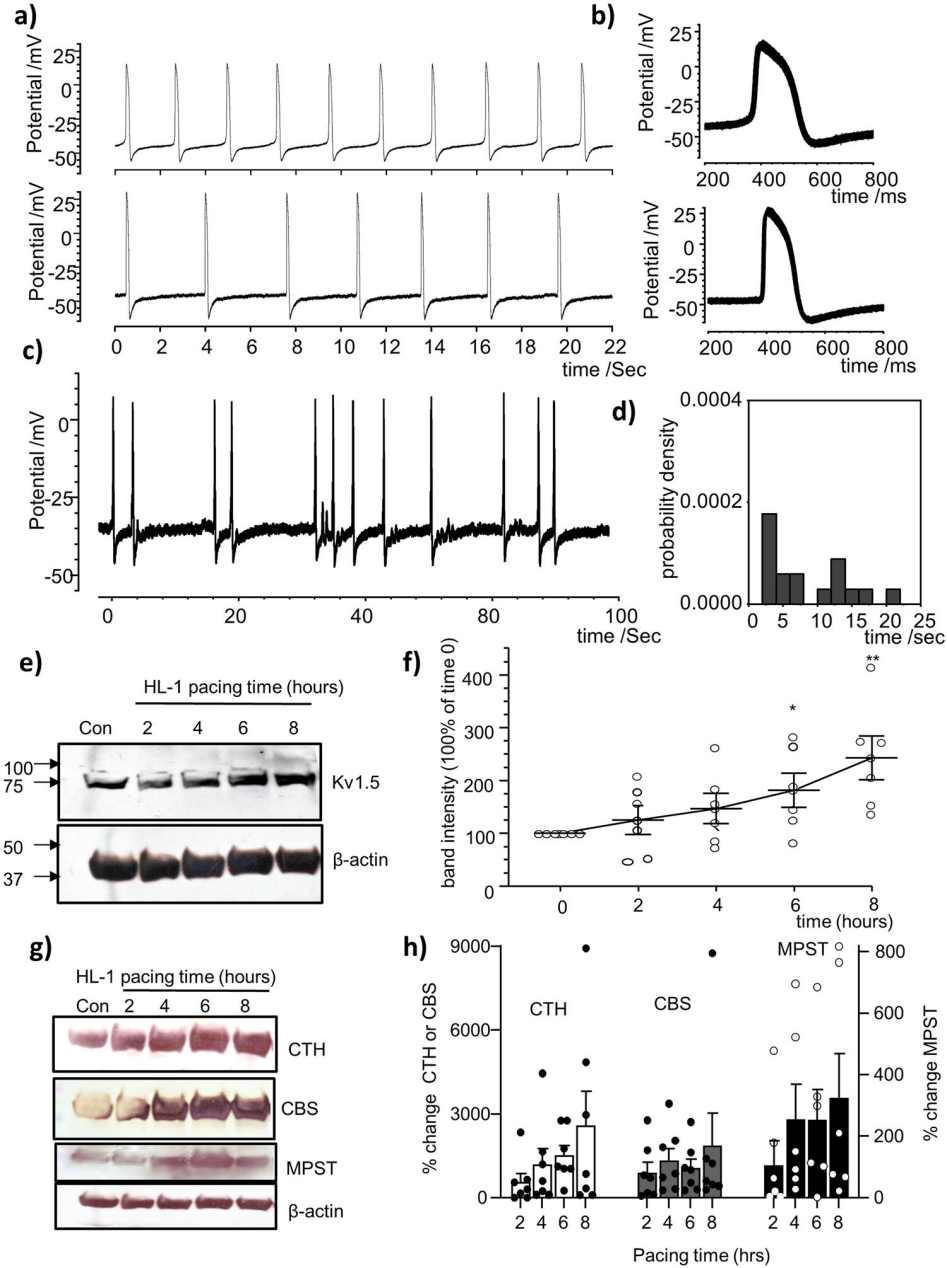
Electrical and cellular remodeling in HL-1 cardiomyocytes via regular interval pacing AP recordings from control and paced HL-1 cardiomyocytes. **a** Representative spontaneous AP recordings from a control unstimulated cell (*upper*) and a cell that had been subjected to high frequency interval pacing (*lower*). **b** Superimposed spontaneous APs (total 36) from a typical control (*upper*) or paced (*lower*) HL-1 myocyte, demonstrating that prior interval pacing results in shortening of spontaneous APs and a decrease in spontaneous AP frequency. **c** Example showing irregularity of intervals between spontaneous APs apparent in some cells after interval pacing. **d** The probability density histograms for these intervals (*N* = 17, 8.6 ± 1.4 sec). **e** Representative western blots for native I_Kur_/Kv1.5 after various interval pacing time periods (0–8 h) showing increased protein level when compared to control unstimulated conditions. **f** Summary mean (±s.e.m) band intensities as determined using ImageJ software (*n* = 6–7 experiments, one-way ANOVA with post hoc Bonferroni correction). **g** Representative western blots for H_2_S producing enzymes at different pacing time periods (0–8 h) showing increased levels of CTH, CBS and MPST when compared to controls (time 0). **h** Summary mean (±s.e.m) percentage change of band intensities (*n* = 6–7 experiments). β-actin was used as loading control in all western blots. For all panels *P < 0.05, **P < 0.01, ***P < 0.001.

To study remodeling in vitro a high frequency (5 Hz) interval pacing protocol was applied to HL-1 cells in culture (for 8 h) before measurements of cellular or electrical electrophysiological parameters. Interval pacing resulted in electrical remodeling of spontaneous APs when compared to control non-paced cells. APD was measured at 50% (APD_50_) and 90% (APD_90_) of repolarization. The mean APD was reduced by approximately 30% at APD_50_ and 35% at APD_90_; from 139.2 ± 8.3 ms to 96.9 ± 7.2 ms for APD_50_ and from 306.9 ± 37.6 ms to 196.0 ± 22.0 ms for APD_90_ (*p*-value = 0.001 and 0.0198 respectively; two-tailed unpaired *t* test *n* = 10). Figure 1a, shows representative spontaneous APs recorded in control non-paced HL-1 cell (upper) and in cell that had been subjected to 5 Hz interval pacing (lower). Figure 1b shows superimposed APs from the cells in Fig. 1a, to illustrate the intrinsic variability. In control unstimulated cells, the APD_90_ had a coefficient of variation of 4.1% and its rhythmic activity had a cycle length of 2.3 ± 0.02 s (*N* = 44), while the cell previously subjected to pacing had a coefficient of variation of 4.2% and a cycle length of 3 ± 0.07 s (*N* = 36). In addition, irregular, short bursts of spontaneous activity were recorded in some cells after pacing (4 out of 10) as illustrated in the example shown in Fig. 1c with irregularity of intervals between spontaneous APs illustrated by a histogram (Fig. 1d) estimating the probability density for these intervals (*N* = 17, 8.6 ± 1.4 s). Such bursts were not observed in control unstimulated cells.

The time course of the cellular changes was assessed using western blot analysis following pacing over an 8-hour period with measurements taken every 2 h. In paced cells, Kv1.5/I_kur_ expression increased as a function of time Fig. 1e. The Mean ± SEM of the normalized cumulative data is shown in Fig. 1f where the value at each time point is expressed relative to the measurement in unstimulated controls. Moreover, H_2_S-generating enzymes CTH, CBS and 3MPST, were all detected in HL-1 cells (Fig. 1g) and the expression of these enzymes increased as a function of time during pacing (Fig. 1g, h and Supplementary Fig 2). The full blots of these can be found as a Supplementary Fig 1.

The use of diphenyl phosphine oxide-1 (DPO-1), a potent inhibitor of Kv1.5^45^, on paced HL-1 resulted in time dependant downregulation of Kv1.5 and to a lower degree the down regulation of H_2_S-generating enzymes (Supplementary Fig 3).

### H_2_S inhibits recombinant Kv1.5

**Fig. 2:**
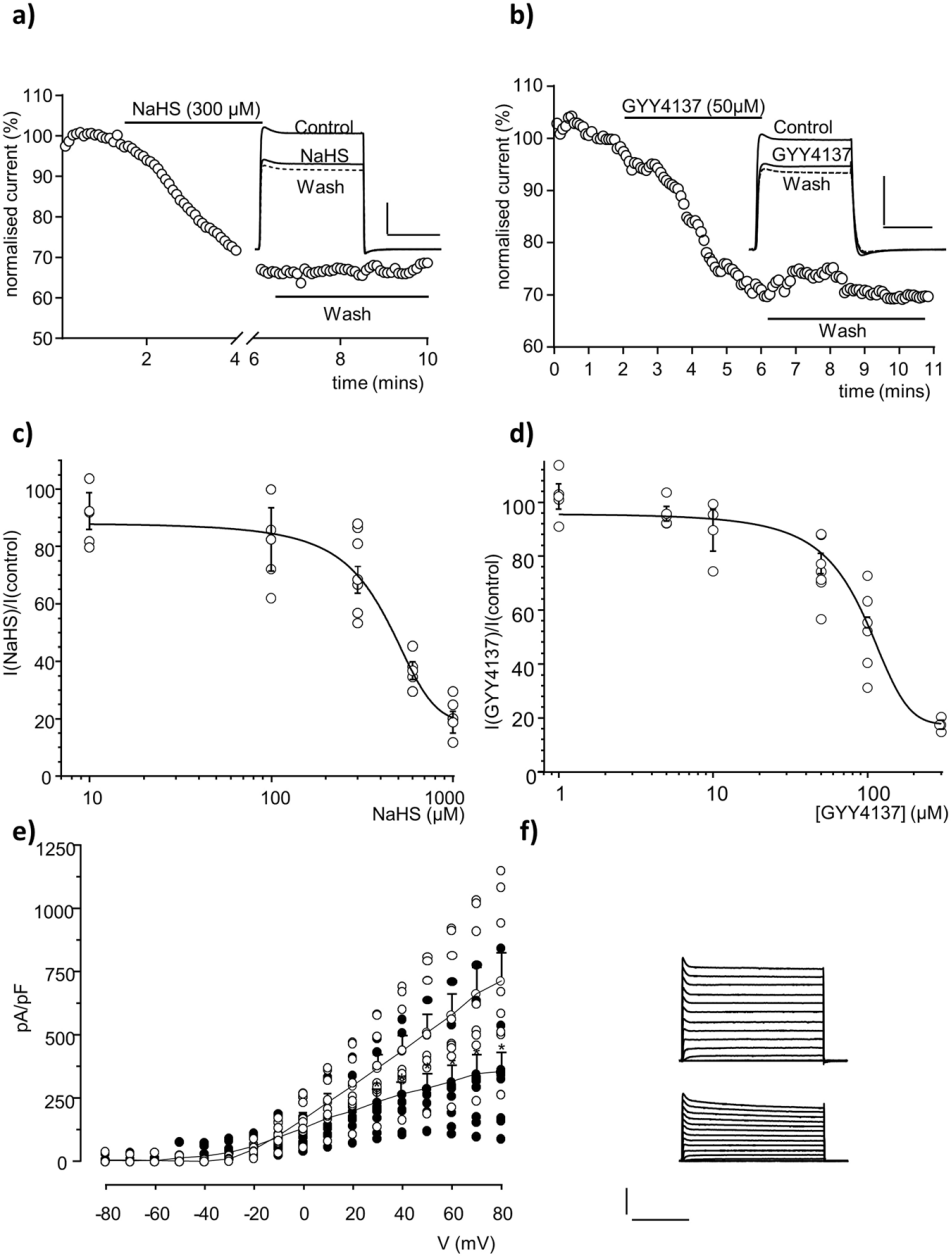
H_2_S inhibits recombinant Kv1.5. **a**, **b** Examples of time-series plots illustrating normalized peak current amplitudes evoked by step depolarizations from −70mV to +50 mV in HEK293 cell expressing hKv1.5 before, during (horizontal bar) and after application of the fast-releasing H_2_S donor NaHS. or the slow-releasing H_2_S donor GYY4137 via perfusate. Insets show example currents under the conditions described; scale bars: 2 nA (*vertical*) and 50 ms (*horizontal*). **c**, **d** Mean ( ± s.e.m., values from 4–7 cells), concentration-response relationship illustrating the effects of H_2_S donor NaHS or GYY4137 on hKv1.5 expressed in HEK 293 cells. Inhibition was determined using currents evoked by step-depolarizations from −70mV to +50 mV. Curves obtained by fitting data to Boltzmann function. **e** Mean ( ± s.e.m.; *n* = 9; *P < 0.05) current-density versus voltage relationships before (open circles) and during (solid circles) exposure to NaHS (300 µM). **f** Families of currents evoked in a HEK293 cell stably expressing hKv1.5 before (*upper*) and during exposure to NaHS (300 µM; *lower*). Currents evoked by step-depolarizations, applied up to +80 mV in 10 mV increments from a holding potential of −70 mV. Scale bars apply to both families of currents 2 nA (vertical) and 200 ms (horizontal).

To examine the modulation of Kv1.5 currents by H_2_S in isolation, HEK293 cells expressing human Kv1.5 were continually perfused during a whole cell patch clamp recording as previously described^41^. The effect of H_2_S on hKv1.5 currents is shown in Fig. 2a, under control conditions before H_2_S was added, the normalized peak amplitude of the current time series evoked by a step despoliation from −70 to +50 mV was steady, with a coefficient of variation of 1.59%. Switching the perfusate to one containing freshly prepared H_2_S-forming compound NaHS, led to a slowly developing inhibition of hKv1.5 currents, which, was not reversed by a wash out. H_2_S inhibited hKv1.5 currents by 29.8 ± 3.1% (300 µM; *n* = 10). Similar results were obtained when hKv1.5 expressing cells were exposed to a second H_2_S-forming compound, GYY4137. A time series example is shown in Fig. 2b; before GYY4137 perfusion and up to 2 min, the measured normalized peak was steady, with a coefficient of variation of 1.66%. Following GYY4137 perfusion there was a slow gradual inhibition of the peak, by approximately a third; this inhibition was not reversed by 5 min wash out (Fig. 2b). The measured peak inhibition of hKv1.5 currents for all examined cells was 25.3 ± 1.4% (50 µM GYY4137; *n* = 6). For these studies, GYY4137 was pre-dissolved and added to the reservoir for at least 30 min, as it is known to release H_2_S gas quite slowly. The inhibition of hKv1.5 by NaHS and GYY4137 was concentration-dependent, with EC values of 383.6 ± 30.5 µM and 91.3 ± 3.01 µM respectively measured at time point of 4 min (Fig. 2c, d). Control experiments for the examined duration, showing normalized hKv1.5 current amplitude can be found in Supplementary Fig 4a.

These observations indicate that H_2_S, derived from two distinct sources, irreversibly inhibits hKv1.5 currents. Currents were inhibited over a wide range of activating test potentials, exhibiting significant difference in their size with test potentials over +30 mV (Fig. 2e) but without significant alteration of channel kinetics in these examined test pulses (Fig. 2f and Supplementary Fig 4b).

### H_2_S inhibits native Kv1.5 in HL-1 cells and prolongs APD in atrial cardiomyocytes

**Fig. 3:**
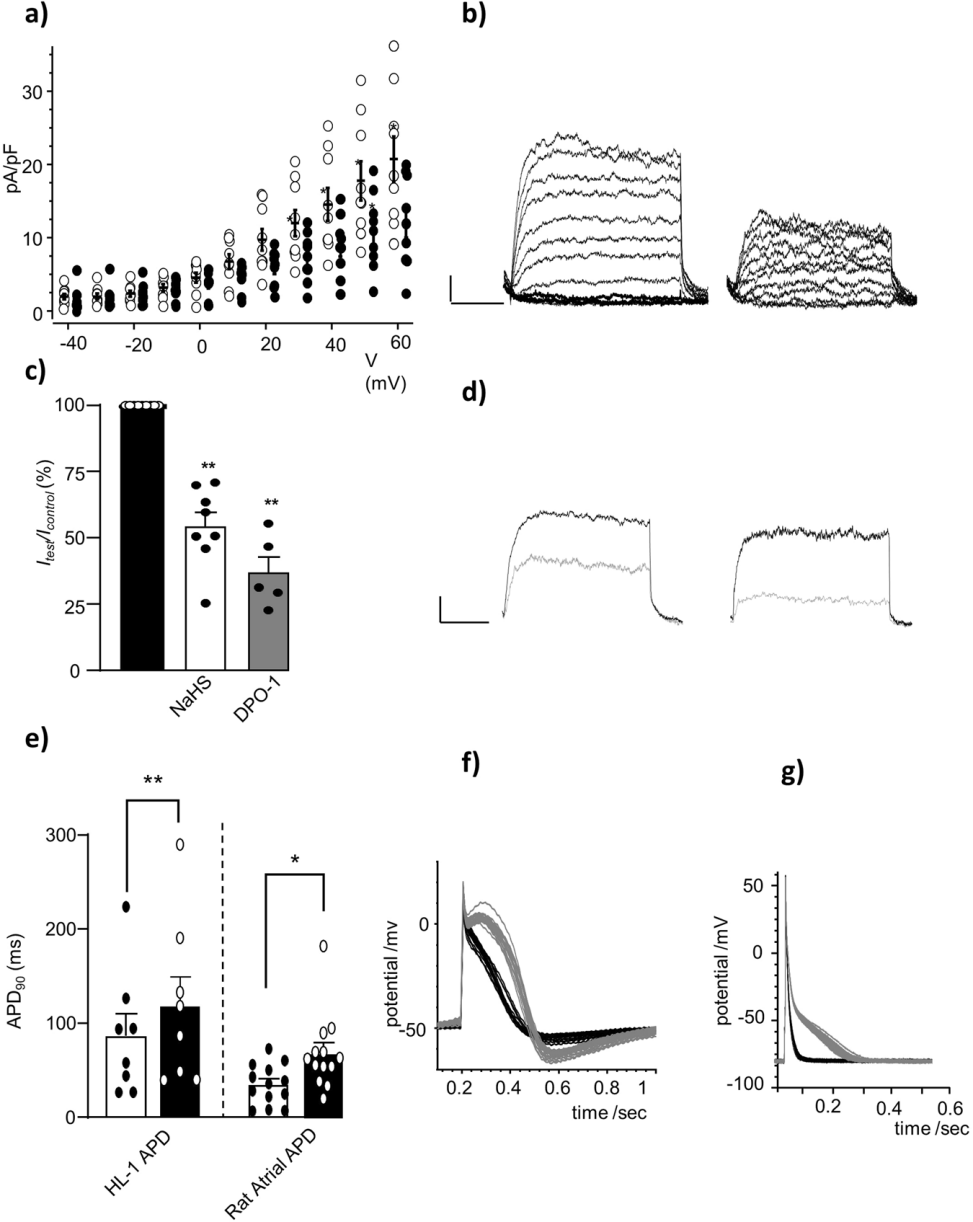
H_2_S inhibits native Kv1.5/ I_Kur_ currents in HL-1 and resulted in prolongation of AP. **a** Current-density versus voltage relationships before (*open circles*) and during (*solid circles*) exposure to H_2_S-releasing donor NaHS (300 µM; *n* = 8, mean ± s.e.m.). **b** Families of outward K^+^ currents recorded in an example HL-1 cell, before (*right*) and during exposure to H_2_S (*left*, 300 µM). **c** Bar graph showing percentage normalized mean (±s.e.m.) effect of NaHS (300 µM) and Kv1.5 inhibitor DPO-1 (1 µM) on outward K^+^ currents peak amplitude measured at +50 mV. **d** Examples showing control recordings (*black*) and the effect of H_2_S (grey, *left*) or DPO-1 (grey, *right*). Scale bars apply to all families of currents 0.1 nA (vertical) and 50 ms (horizontal). **e** Cumulative APD_90_ data (mean, ± s.e.m,) recorded from HL-1 cells or in adult rat atrial cells before (*control, unfilled*) and during application of 300 μM NaHS (*filled*). **f** Superimposed evoked APs (*N* = 20) before (*black*) and during 100 µM NaHS (*grey*) perfusion in HL-1 cells or (**g**) in rat atrial myocyte. For all panels paired *t* test; *P < 0.05, **P < 0.01.

Figure 2 shows that H_2_S inhibits hKv1.5 currents in HEK293 cells expressing the recombinant channels. To further assess whether H_2_S may also inhibit native I_Kur_ currents, (generated primarily via Kv1.5 in the atrial tissue), we examined its effect in HL-1 cells where the role I_Kur_ plays in the AP is more pronounce. Using protocol to functionally isolate I_Kur_^41,46^, it was found that outward K^+^ currents generated were, like recombinant currents, inhibited by application of H_2_S generated via the NaHS donor. This inhibition occurred over a wide range of activating test potentials (Fig. 3a, b). The inhibitory effect of H_2_S on Kv1.5/I_Kur_ at +50 mV test potential was similar to that observed for recombinant channels (38.3 ± 7.2 and 29.8 ± 3.1% respectively). These outward K^+^ currents were also strongly inhibited when DPO-1 was used (Fig. 3c, d). Further evidence of the contribution of Kv1.5/I_Kur_ to the K^+^ outward currents obtained via applying H_2_S followed by DPO-1. Using this approach, H_2_S caused an inhibition of the K^+^ current with cumulative mean inhibition of 31.8% ± 4.07% (*n* = 5), measured at +50 mV test potential, close to that seen above (29.8 ± 3.1%). Following the perfusion of DPO-1 and in the presence of H_2_S, there was a further small inhibition of 8.8% (*n* = 5), although this was not statistically significant (Supplementary Fig. 5d, e).

Moreover, under current clamp conditions, we assessed the effect of H_2_S on evoked APs in HL-1 atrial cells (Fig. 3f) and in adult rat (Fig. 3g) isolated atrial cardiomyocytes, where Kv1.5 expression has been established^47^. Measured APD_90_ significantly increased in both cell types; from 86.9 ± 23.2 to 118.7 ± 30.7 ms (*n* = 8, *p*-value = 0.006; two-tailed paired *t* test) in HL-1 cells with no significant change in membrane potential before (−61.6 ± 3.6 mV) or during (−63.1 ± 4.3 mV) exposure to H_2_S, and from 33.5 ± 6.09 to 69.7 ± 12.1 ms (*n* = 13, *p*-value = 0.007; two-tailed paired *t* test) in isolated rat atrial myocytes, with no significant change to membrane potential before (−78.9 ± 1.9 mV) or during (−75.4 ± 1.9 mV) exposure to H_2_S, (*n* = 13). Similar effects on APD in isolated rat atrial myocytes were seen when DPO-1 was used (Supplementary Fig. 5a). Taken together these data suggest that H_2_S can act as a regulator of this channel in these atrial tissues.

### Cellular remodeling stringency is reduced when H_2_S is present during pacing HL-1 cardiomyocytes

**Fig. 4:**
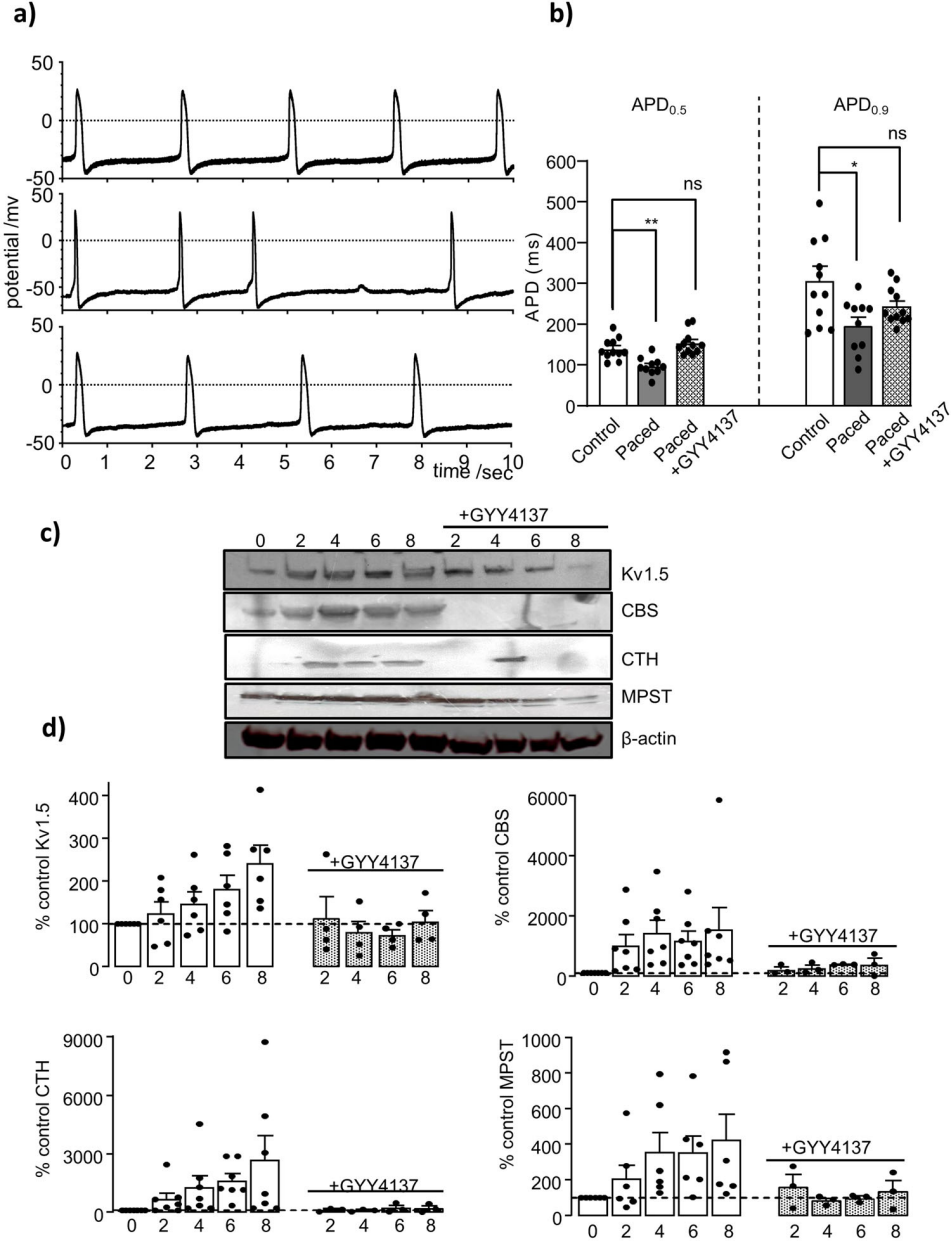
H_2_S reduced electrical and cellular remodeling in paced HL-1 atrial cardiomyocytes. **a** Representative AP recordings in spontaneously beating HL-1 cells from control cells that were not paced during the 8 h culture period (*upper*), cells that were paced at 5 Hz for 30 min regular intervals (*middle*) and paced cells that were incubated with slow-releasing H_2_S donor GYY4137 (*lower*). **b** Cumulative data showing mean (±s.e.m.) APD under control conditions (*n* = 11), in paced cells (*n* = 10), and in paced cells incubated with GYY4137 (*n* = 11).*P < 0.05, **P < 0.01. **c** Representative western blots for native I_Kur_/Kv1.5, and H_2_S producing enzymes CBS, CTH and MPST at different pacing time periods (2–8 hr) in the absence of GYY4137 or with incubation with GYY4137 (horizontal line), compared to control unstimulated conditions (0 h). β-actin was used as loading control in all western blots. **d** Mean (±s.e.m) percentage change of band intensities following (*n* = 4 experiments) at different pacing time periods (2–8 hr) in the absence of GY4137 or with GYY4137 incubation, as indicated by the horizontal line.

Given the reported cardioprotective role of H_2_S^48,49^, we proceeded to examine its effect on paced HL-1 cells incubated with the H_2_S slow-release molecule, GYY4137. Samples were collected at various time intervals and compared to paced cells without the addition of H_2_S for a total of examined pacing period of 8 h. The electrophysiological remodeling observed in HL-1 cardiomyocytes, characterised by shortening of the spontaneous APs after high frequency pacing in culture, was reduced when H_2_S was present in the media during pacing. Figure 4a, shows spontaneous APs recorded from a control unstimulated cell (upper), paced cell (middle), and a cell where H_2_S was present throughout the pacing protocol (lower). Importantly, there was no significant difference in APD between control cells and paced cells that were supplemented with H_2_S donor GYY4137 (Fig. 4b). The mean ± SEM of APD_50_ and APD_90_ values for control unstimulated cells were 139.1 ± 7.1 ms and 306.9 ± 35.9 ms (*n* = 11) and for paced cells where H_2_S was present throughout the pacing protocol were 153.6 ± 8.6 ms and 244.3 ± 13.76 ms (*n* = 11; *p*-value = 0.277 and 0.117 respectively; two-tailed unpaired *t* test).

We also investigated the effect of H_2_S on the pacing induced changes in the expression of Kv1.5/I_Kur_ and H_2_S-producing enzymes (Fig. 1e–h) as shown in the western blot examples, the upregulation of Kv1.5 and the H_2_S-producing enzymes, CBS, CTH, and 3MPST were reduced by the presence of the H_2_S donor GYY4137 during high frequency pacing (Fig. 4c), full blots of these can be found as a Supplementary Fig 6. Figure 4d shows the quantified mean ± SEM of the cumulative data for paced cells with and without H_2_S, the presence of H_2_S effectively prevented the pacing-induced increase in expression of Kv1.5/I_Kur_ and H_2_S-producing enzymes (relative to unstimulated cells) and when compared to DPO-1 GYY4137 appear more effective in reducing/preventing CBS and CTH (Supplementary Figs. 3 and 6). The prevention of Kv1.5/I_Kur_ upregulation by H_2_S during the pacing period may explain (i) AP durations in these cells remained closer to the unstimulated controls and (ii) the reduced upregulation of H_2_S-producing enzymes.

### Inhibition of Kv1.5 by H_2_S requires NO formation and arises through S-nitrosylation

**Fig. 5:**
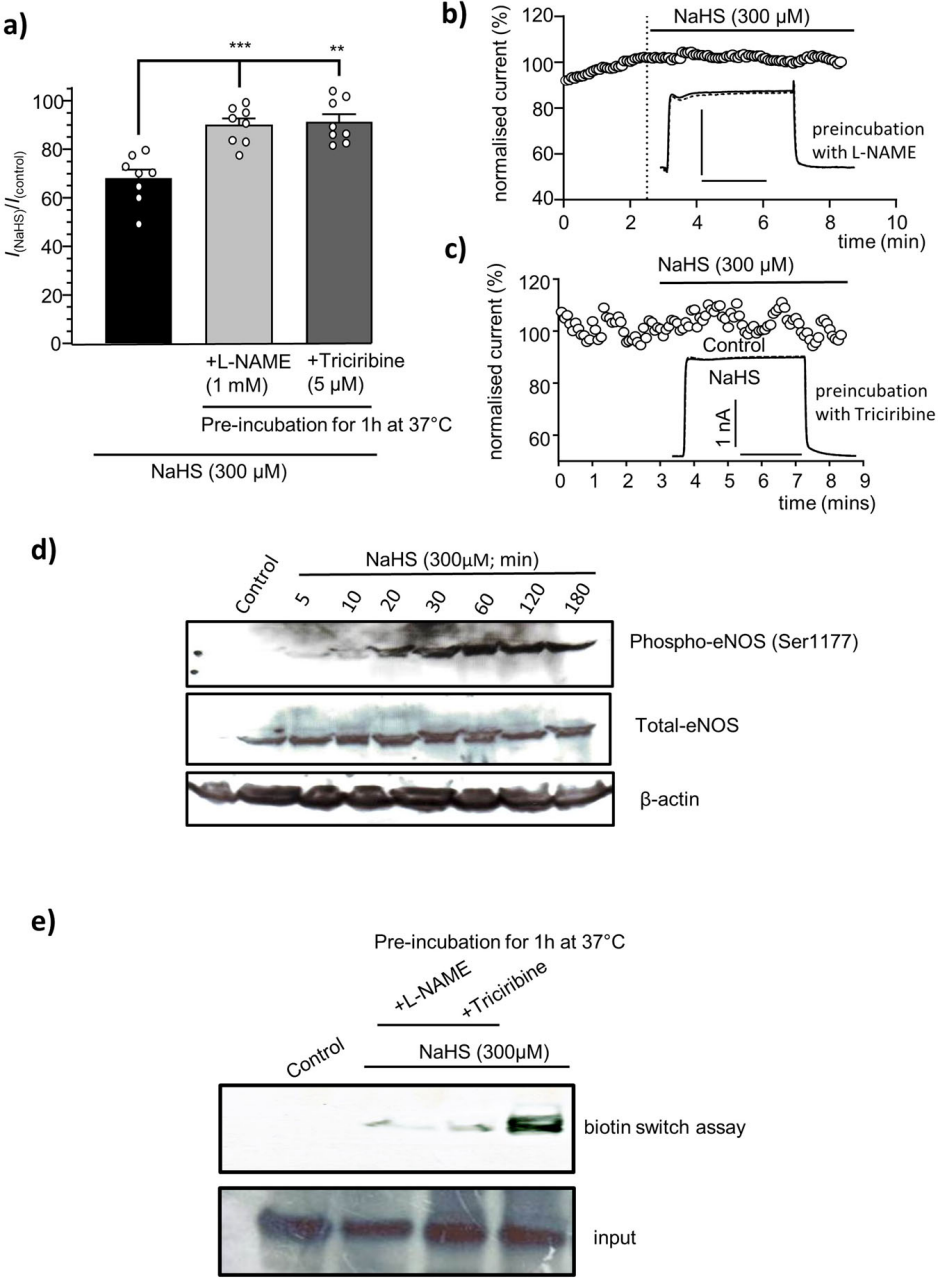
Inhibition of Kv1.5 by H_2_S requires NO formation and nitrosylation of hKv1.5. **a** Cumulative data showing the percentage inhibition (mean ± s.e.m.) induced by the H_2_S donor NaHS on normalized hKv1.5 peak amplitude in HEK293, currents evoked by repeated step depolarizations from −70 mV to +50 mV (100 ms duration, 0.2 Hz) in untreated (*n* = 8), L-NAME (1 mM; 1 h, 37 °C; *n* = 8) or triciribine pre-treated cells (5 µM; 1 h, 37 °C; *n* = 8) **P < 0.01, ***P < 0.001. **b**, **c** Example time-series plots showing the lack of effect of H_2_S donor NaHS during perfusion on normalized peak current amplitudes obtained from either L-NAME or Triciribine pre-treated HEK293 cell stably expressing human Kv1.5 (hKv1.5). Inset shows example currents before and during NaHS exposure (horizontal scale bar = 50 ms). **d** Representative western blots showing the time dependent effect of NaHS treatment on eNOS phosphorylation at Ser 1177 (*upper*), eNOS expression (*middle*). β-actin used as loading control (*lower*). Cell lysates obtained from H_2_S treated HEK293 cell stably expressing hKv1.5 at the time indicated, control represent cells not exposed to H_2_S. **e** Western blot showing detection of Kv1.5 nitrosylation via the biotin-switch assay.

H_2_S modulates target proteins, including ion channels, via several distinct mechanisms^37^, and in the cardiovascular system the reduced bioavailability of NO is associated with cardiac dysfunction, in particular, AF^25^ and heart failure^50^. To investigate the potential involvement of NO in the inhibition of Kv1.5 by H_2_S, we examined its effects in HEK 293 hKv1.5-expressing cells that had been pre-exposed to L-NAME (1 mM, 1 h at 37 °C), to inhibit NO formation via the inhibition of NO synthase, and triciribine, to prevents NO formation via inhibition of Akt^51^ (5 µM, 30 min at 37 °C). As illustrated in Fig. 5a, the inhibition of NO formation prevented inhibition of hKv1.5 by H_2_S applied via NaHS (300 µM). Time series examples shown in Fig. 5b and in Supplementary Fig. 7c, with cells pre-exposed to either L-NAME or triciribine.

In control conditions and before the perfusion of H_2_S via NaHS, the measured currents amplitudes in both cells were stationary with coefficient of variation of 4% in Fig. 5b where the cell was pre-treated with L-NAME, and 4.3% in Fig. 5c where the cell was pre-treated with triciribine, and 4.2% (Fig. 5b) or 0.8% (Fig. 5c) following the perfusion of NaHS and measured during the last 2.5 min of the recording. Furthermore, H_2_S treatment of HEK 239 hKv1.5-expressing cells resulted in a time dependent increase in phosphorylation of eNOS at position Ser1177 (Fig. 5d), the full blots can be found as a Supplementary Fig 7a, eNOS has been shown to regulate NO synthesis by activation of phosphorylation at this position^52,53^. These findings suggest NO is required for H_2_S to inhibit Kv1.5. In further support of this requirement, previous studies have demonstrated that recombinant Kv1.5 is inhibited by the NO-donor S-Nitroso-N-acetyl-DL-penicillamine^40^ (SNAP; 100 µM; *n* = 4), we have confirmed this finding, in close agreement with the earlier study with inhibition of 15.8 ± 5.2%.

Since S-nitrosylation is considered to play a role in cardioprotection^54,55^ we examined this role further using the biotin switch assay^56,57^. H_2_S exposure via application of NaSH led to S-nitrosylation of the hKv1.5 channel, this modification was reduced in HEK 293 hKv1.5 expressing cells that were pre-treated with L-NAME for 1 h or triciribine for 30 min before and during H_2_S exposure (Fig. 5e), the full blots can be found as a Supplementary Fig 7b.

## Discussion

Here we provide the modulation of Kv1.5/I_Kur_ by H_2_S, and propose a role for this gasotransmitter in protecting against cellular and electrophysiological remodelling produced by high frequency pacing. Protection occurs via the inhibition of the atrial ultra-rapid outward rectifying current (I_Kur_) and its underlying Kv1.5 channel. HL-1 cardiomyocytes have previously been used successfully for the in vitro modelling of AF^9,58,59^ with high frequency pacing reproducing many features of in vivo tissue remodelling. Some of these features have been shown to be mediated by oxidative stress^60^.

In the present study, interval pacing has resulted in electrophysiological remodeling of HL-1 cells, including irregular activity and a significant APD shortening (Fig. 1). The APD shortening is a common characteristic of atrial remodelling in AF^12^, and here it was observed in all examined cells subjected to high frequency pacing. This would be expected to make cardiac tissue substrate more capable of sustaining a re-entrant arrhythmia^61^. Within cardiac tissue, cells with such high frequency irregular bursting could act as a trigger for arrhythmia initiation.

Another characteristic of AF remodeling is the altered tissue expression of ion channels^3,13^. We show an enhanced expression of Kv1.5/I_kur_ following the interval pacing conditions used in this study (Fig. 1e). This upregulation would contribute to the shortening of APD and creating a reduced effective refractory period, thus increasing the likelihood of re-entrant arrhythmias. Increased expression of Kv1.5 in rat cardiomyocytes has been implicated in development of arrhythmias and also caused APD shortening^62^.

In addition, we also observed the upregulation of protein expression of the H_2_S-producing enzymes; CTH, CBS and 3MPST (Fig. 1g). The increased production of such enzymes might act as a feedback mechanism, accelerating an increase in the cellular levels of H_2_S and consequently, offering a reduction in the remodeling effects. Such a protective role has been proposed by several studies in various diseases, including cardiovascular disease^35^. H_2_S is a modulator of diverse cardiovascular functions; it provides cardioprotection against ischemia/reperfusion (I/R) injury^63,64^ and it causes vasodilation through K_ATP_ activated channel sulfhydration and activation^43^. However, its acute vascular effects also include an endothelium-dependent component^65^, which may involve NO production or activity^66^. Most strikingly, the deletion of H_2_S-producing enzyme CTH resulted in hypertensive mutant mice, implicating H_2_S as having a role as a physiological regulator of blood pressure^42^. Although H_2_S has been demonstrated to be effective in the regulation of coronary circulation, its importance is not fully established^65^, in particular its involvement in coupling flow to metabolism has yet to be explored.

Class I and III anti-arrhythmic agents, act on Nav1.5, to prolong the AP, but are only partially effective at restoring normal atrial rhythm, and can trigger life threatening ventricular arrhythmias^30^. Therefore, current attention in pharmacological management of AF is focused on identifying selective targeting of cardiac ion channels which are expressed mainly in the atria, including Kv1.5^67,68^. Results to date are promising, as Kv1.5 inhibition restores atrial contractility in animal models of AF^29,69,70^. This occurs even though its functional expression is reduced in chronic AF, and despite the complexity of remodelling in AF^71–73^. Targeting the oxidative regulation of Kv1.5 (either its acute oxidative augmentation of activity or controlling its oxidant-induced internalization/degradation) represents a novel and potentially important strategy for regulating its functional expression and activity as an approach to the management of AF.

Examination of the modulation of Kv1.5 by H_2_S, both in isolation and in established model of AF (Figs. 2 and 3) demonstrate that H_2_S inhibits stably expressed Kv1.5 in HEK293 cells. This direct inhibition was seen using whole cell patch clamp recordings as a slow progressive inhibition reaching a steady-state within 4–5 min and it was not reversed (Fig. 1). Combination of both perfused H_2_S and NO generated within the cell may contribute to the irreversible effect observed here. Interaction between H_2_S and NO modulation mechanism is essential in their regulatory action^74–78^. For example, sulfanyl (HS^•^) and nitrogen monoxide (^•^NO) chemical interaction generate thionitrous acid^79^ (HSNO), in turn this would produce bisulfide (HS^−^) that could alter protein function when nitrosating biomolecules present.

H_2_S also inhibited K^+^ currents in HL-1 cells, these K^+^ currents were extremely sensitive to the selective Kv1.5 blocker DPO-1, consequently, the inhibitory effect of H_2_S on these outward K^+^ currents is likely to be predominantly on the activity of Kv1.5 in these cells (Fig. 3a–d and Supplementary Fig. 5d, e). Additionally, APs in HL-1 cells were elongated when H_2_S was applied (Fig. 3e, f and Supplementary Fig. 5c). These cells exhibit some APs with spontaneous diastolic depolarization phase. Spontaneous depolarization is often due to hyperpolarization activated current (HCN), shown to be expressed in these cells^80^. The HCN current appears to be enhanced by nitric oxide^81,82^, accordingly it is reasonable to assume some activation via NO occurring of HCN following H_2_S application (Fig. 3f). However, there are multiple ionic conductances contribute to the diastolic depolarization and dispute as to the relative roles of calcium clock and membrane (potassium delayed rectifier currents deactivation, HCN) mechanisms involved in this diastolic phase^83,84^. While these data do not eliminate the effect of H_2_S on other cardiac ion channels, and indeed several such ion channels are known to be H_2_S-sensitive^43^, the fact that the prolongation of APD by H_2_S in healthy rat atrial myocytes and healthy HL-1 cardiomyocytes significantly mimics the effect of DPO-1 (Supplementary Fig. 5b) emphasizes the role of regulation of Kv1.5.

The proposed regulation of Kv1.5 by H_2_S could potentially be of physiological significance in regulating atrial excitability and supports a putative selective therapeutic role for Kv1.5 inhibition in AF. The supplementation of H_2_S donor GYY4137 into HL-1 cells remodeled by high frequency pacing eliminates or reduces the cellular remodelling changes detected previously (Fig. 4). This is consistence with the cardioprotection effect of H_2_S and adds to the data supporting the importance of H_2_S as a signalling molecule^35,36^. H_2_S donors resulted in oxidative stress reduction and also to the prevention of angiotensin II induced cardiac hypertrophy in rat myocytes^68,85–87^. The particular cellular cardio protective mechanism of GYY4137 remains unknown. However, the release of low concentration of H_2_S by this donor over a long period of time^88^ and thus it is present during the examined period of HL-1 pacing could provide continuous supply of H_2_S that act as a strong scavenger for peroxynitrites^89^ thus eliminating their damaging effect^90^. Moreover, H_2_S allows reduction of disulfide bonds^91^ and could act as a reducing agent when reactive intermediates present, generating products that offer an additional non-enzymatic pathway that results in increased NO bioactivity. These intermediates products include Nitroxyl^92^, nitrosopersulfide^93^ and thionitrous acid^79^.

The inhibition of hKv1.5 channels expressed in HEK293 cells by bath application of two distinct H_2_S donors examined on single cell level (Fig. 2) and the significant reduction of this effect when NO formation was prevented by pre-treatment of cells with L-NAME and Triciribine (Fig. 5) suggesting that H_2_S acts, at least in part, via NO formation. In addition, biochemical measures revealed that H_2_S treatment of HEK293 cells expressing hKv1.5 resulted in a time dependent increased activation of eNOS, a prerequisite enzymatic mechanism for NO production, via phosphorylation of position Ser1177^94^. This phosphorylation was apparent (Fig. 5d) even from 5 min exposure to H_2_S, showing a faint band and reaching peak at time 30 min, demonstrating H_2_S dependent activation of NOS.

Interestingly, maintenance or augmentation of NO bioavailability is also considered a viable approach in the treatment of AF^20,40^. Furthermore, a post-translational modification of cysteine residues by S-nitrosylation, involved in forming –SNO groups by NO, has emerged as an important feature of NO signalling. Enhanced S-nitrosylation levels were detected when H_2_S and NO, applied via SNAP, were both used together during reperfusion of mouse hearts, suggesting that the protective effect of H_2_S was dependent on NO signalling^95^.

Other cardiac channel proteins were found to be regulated by S-nitrosylation as a mechanism to enable cardioprotection, for instance, enhancement of the Na^+^ current^96^ and the inhibition of the α1C subunit of the L-type Ca^2+^ channel^97^. We have detected S-nitrosylation of hKv1.5 using the biotin switch assay following HEK293 cells expressing hKv1.5 treatment with the H_2_S donor, NaHS, once again, this S-nitrosylation was reduced when NO formation was prevented (Fig. 5d). Kv1.5 S-nitrosylation was suggested^40^, but not demonstrated, to occur in the voltage-sensor region of the channel, at either C331 and/or C346 and stabilized by I262 and R342, and thus may effect conformational change of Kv1.5 protein.

Our data indicate that exposure to H_2_S increases NO production through an increased phosphorylation of eNOS at Ser1177. An increase in H_2_S levels may promote a possible AF treatment through increased NO bioavailability and S-nitrosylation as a mechanism for H_2_S-mediated inhibition of hKv1.5.

We provide insight into the influence H_2_S on the modulation of Kv1.5, which is associated with adverse electrical remodelling in AF, the reduced remodeling observed following H_2_S donor GYY4137 supplementation offer a potential therapeutic value for H_2_S being a regulator of the atrial excitability especially as the incidence of AF increases with age^98^ and bioavailability of NO^25^ and H_2_S^99^ decreases.

## Methods

### Cell culture

HL-1 atrial cardiomyocytes (Sigma, UK) were maintained in Claycomb media supplemented with batch specific FBS (10%), norepinephrine (0.1 mM), penicillin/streptomycin (1%), and l-glutamine (2 mM). Cells were cultured in flasks, or on coverslips that has been pre-treated with gelatin from bovine skin (0.02%) and fibronectin (5 µg ml^−1^). HEK293 cells expressing Kv1.5^41^ were maintained in minimum essential medium supplemented with fetal calf serum (10%), non-essential amino acids (1%), antibiotic antimycotic mix (1%) gentamicin (50 μg ml^−1^), and blasticidin (5 μg ml^−1^). All cells were cultured at 37 °C in a humidified atmosphere containing 95% air and 5% CO_2_ and passaged when reached confluency.

### HL-1 pacing

HL-1 cells were seeded onto a four well plates or coverslips and incubated at 37 °C in a humidified atmosphere (95% air and 5% CO2) with media being replaced every 24 h until visible beating was seen under microscopic examination. Cells were then subjected to electrical field stimulation using a C-Pace EM, a multi-channel culture pacer, (IonOptix, Milton, MA, USA), and incubated at 37 °C in a humidified atmosphere (95% air and 5% CO2) for 8 h. To induce tachycardia HL-1 cardiomyocytes were paced at 5 Hz with 5-ms duration square wave (40 V). The protocol lasted for a total of 8 h and pacing was applied for 50% of the time (30 min/hour). In some experiments, where H_2_S effect was examined, GYY4137 (50 µM) was added to the media for the experiment duration. The cellular changes induced by pacing in HL-1 cells were assessed by collecting time course samples at 2, 4, 6 and 8 h and compered to the same cell population that was not subjected to pacing. Cell samples were lysed in M-perTM (Perbio Science, UK) with added tablet of complete mini protease inhibitors (Roche Diagnostics UK, UK). Cell lysate were cleared by centrifugation and stored at −20 °C until needed.

### Isolation of rat atrial myocytes

Wistar rats (150-200 g) were humanely euthanized in accordance with UK Home Office Guidance on the Operation of Animals (Scientific Procedures) Act 1986 and with university of Leeds Ethical Review Committee approval (AWCNRWDS130706). Isolated hearts were perfused with Tyrode solution^100,101^, following the initial collagenase digestion, the atrial tissue was removed and cut into small pieces in a collagenase-BSA containing solution and gently agitated at 37 °C. Atrial cell suspension with rod-shaped cells were collected by filtration through nylon gauze and gentle centrifugation. Cells were washed in Tyrode solution containing 0.4 mM Ca^2+^ and re-suspended in the same solution containing 0.7 mM Ca^2+^. All experiments were executed at room temperature (20–22 °C).

### Electrophysiology

HL-1 cells attached to glass coverslip fragments were placed in a recording chamber (2–4 ml/min; 200 µl volume) on the stage of an inverted microscope (Olympus CK40; Olympus, London, UK). Rat atrial myocytes were allowed to settle for 10 min before perfusion then perfused with extracellular solution (mM; 140 NaCl, 4 KCl, 1.8 CaCl_2_, 1 MgCl_2_, 5 glucose, 10 HEPES, pH 7.4). Electrodes were filled with internal solution (mM; 140 KCl, 10 NaCl, 4 MgCl_2_, 20 EGTA, 10 HEPES, pH 7.2), and the resistance was between 4~6 MΩ when filled. An agar bridge was made of 3 M KCl and used to stabilise the reference electrode when H_2_S was applied. Whole cell configuration for voltage or current clamp experiments were established, recorded, digitized and stored with an Axopatch 200B amplifier, Digidata 1322 A and pCLAMP 10 respectively (Molecular Devices, Union City, CA, USA).

Recordings from HEK293 cells expressing human Kv1.5 or native I_Kur_ from HL-1 cardiomyocytes were obtained using established protocols^41,46^. Action potentials (APs) were recorded either in gap-free mode for the Spontaneous AP or through current injections to trigger them. Signals were low pass filtered at 2 kHz and sampled at 10 kHz. All recordings were done at room temperature. For H_2_S application Sodium hydrosulfide (NaHS; Sigma-Aldrich, Dorset, UK) was freshly made on the day of the experiment and dissolved in water to make 100 mM stock solutions, further dilutions were made in the perfusate solution to the desired test concentrations. GYY4137 Dichloromethane complex (Sigma-Aldrich, Dorset, UK), is a donor slowly releasing H_2_S over time, was prepared as instructed by the supplier and used for experiments that required a longer incubation time.

### Biotin-switch assay

S-nitrosylation was detected using biotin-switch assay^56,57^. HEK293 cells expressing hKv1.5 were grown in T75 flasks until near confluency before being treated with freshly prepared NaHS (300 µM) for 30 min at 37 °C. For experiments that involved L-NG-nitroarginine methyl ester (L-NAME; 1 mM) or Triciribine hydrate (5 µM), cells were preincubated with either drug for 1 h at 37 °C before NaHS treatment. Control untreated cells were grown in separate incubator to that of NaHS experiments to avoid H_2_S gas contamination. Cells were lysed in a non-denaturing lysis buffer (in mM: Tris-HCl 50, NaCl 300, EDTA 5, and Triton-X 1%), protease inhibitor cocktail tablets were added to all buffers used (Roche, Welwyn Garden City, UK). The following steps performed in the dark; to make free thiols unreactive, methyl methanethiosulfonate (MMTS) was used, then, nitrosothiols were selectively reduced with sodium ascorbate (1 mM) to reform thiol and labelled using biotin (biotin-HPDP, Pierce, Loughborough, UK), Biotinylated proteins were then purified using avidin-affinity chromatography, subjected to SDS-PAGE and detection via western blotting according to the manufacture’s instruction (Bio-Rad, UK).

### Immunoblotting

Protein concentrations in cleared cell lysates were determined using a BCA assay (Pierce, USA). Samples (30 μg protein) were loaded on precast mini-protean gels 4–20% gradient and subjected to electrophoresis then transferred to PVDF membranes according to the manufacturer’s protocols (Bio Rad, Hertfordshire, UK). Membranes were blocked with 5% non-fat milk protein in TBS-Tween (TBS, 0.05%) for 1 h at room temperature and immunostained with the appropriate antibodies (1:1000 dilution; anti-CBS, Santa Cruz Biotechnology, UK, anti-Kv1.5 antibody, Neuromab, Davis, CA, anti-CTH or anti-3MPST, Sigma-Aldrich, Dorset, UK) in 1% non-fat milk protein in TBST (1 h at room temperature). Membranes were then washed (3 × 5 min in TBST) and afterward incubated with secondary antibody conjugated to horseradish peroxidase (anti-rabbit Ig for 3MPST and CTH or anti-mouse Ig for anti-Kv1.5 or CBS, both 1:5000 dilution; LI-COR Biosciences, UK) for 1 h at room temperature. Finally, membranes were again washed in TBST (3 × 5 min) and immunoreactive bands visualized using the enhanced chemiluminescence (ECL) detection system (LI-COR Biosciences, UK). Band intensities were measured using ImagJ analysis software.

### Statistical information

Data were analyzed using Excel (Microsoft, UK) and Origin (Northampton, MA) software. Data are presented as mean ± SEM. For statistical comparisons Student’s *t* tests or one-way ANOVA with Bonferroni’s multiple-comparison test was used, with *p* < 0.05 taken as statistically significant in each case. ‘*n*’ indicates the number of cells and ‘*N*’ the number of APs.

### Reporting summary

Further information on research design is available in the Nature Portfolio Reporting Summary linked to this article.

## Supplementary information

Supplementary Information

Description of Additional Supplementary Files

Supplementary Data 1

Reporting Summary

## Data Availability

The original contributions presented in the article/Supplementary figures and as Supplementary data are included in this study, any further inquiries can be directed to the corresponding author.
