# Supplementary material for: Inhibition of the voltage-gated potassium channel Kv1.5 by hydrogen sulfide attenuates remodeling through S-nitrosylation-mediated signaling
Source: Commun Biol. 2023 Jun 19;6:651. doi: 10.1038/s42003-023-05016-5 (PMC10279668; doi:10.1038/s42003-023-05016-5)
Supplement: Supplementary file 1 — Supplementary Information [file 42003_2023_5016_MOESM1_ESM.pdf]

Supplementary Figures

INHIBITION OF THE VOLTAGE-GATED POTASSIUM CHANNEL KV1.5 BY HYDROGEN SULFIDE ATTENUATES REMODELING THROUGH S-NITROSYLATION-MEDIATED SIGNALING

Moza M. Al-Owais, Nishani T Hettiarachchi, Mark L. Dallas, Jason L. Scragg, Jon Lippiat, Arun V. Holden, Derek S. Steele & Chris Peers.

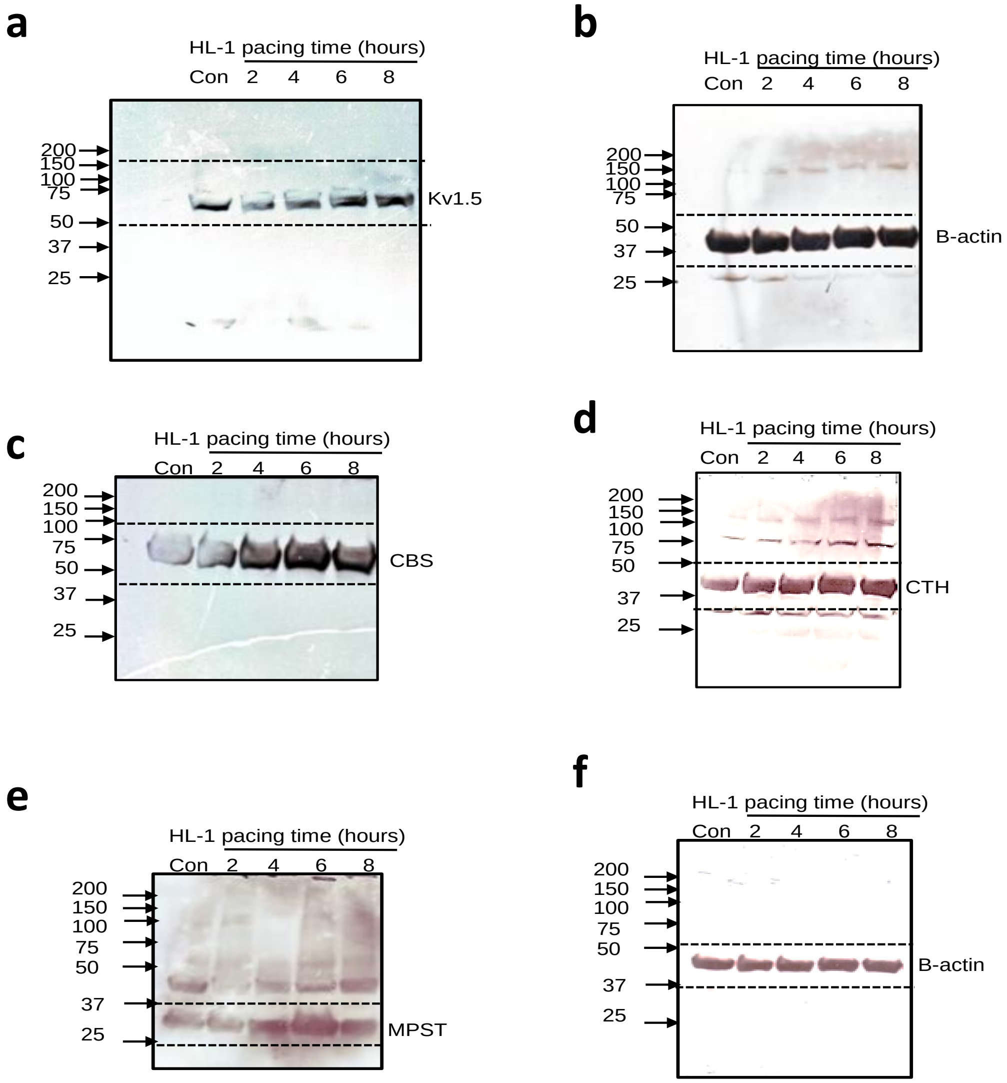

Supplementary Fig 1. Full Western Blots corresponding to Fig 1 e and g, cellular remodeling in HL-1 atrial cardiomyocytes via regular interval pacing full blot scans with cropped lines as indicated by dashed lines. The following antibodies were used for detection; anti-Kv1.5 potassium channel, clone K7/45 (UC Davis/NIH NeuroMab Facility) with expected molecular weight of approximately 70 kDa band (**a**) and loading control β-actin (**b**; Sigma-Aldrich). Cystathionine β-synthase antibody (CBS; Santa Cruz Biotechnology) with expected molecular weight of approximately 63 kDa band (**c**). Cystathionine γ-lyase antibody (CTH; Sigma-Aldrich) with expected molecular weight of approximately 45 kDa band (**d**). Mercaptopyruvate sulfurtransferase antibody (MPST; Sigma-Aldrich) with expected molecular weight of approximately 33 kDa band **(e)** and the loading control β-actin (**f** ; Sigma-Aldrich). HL-1 Cell lystae was obtained from paced cells at time indicated with horizontal bar, control corresponds to unstimulated cells.

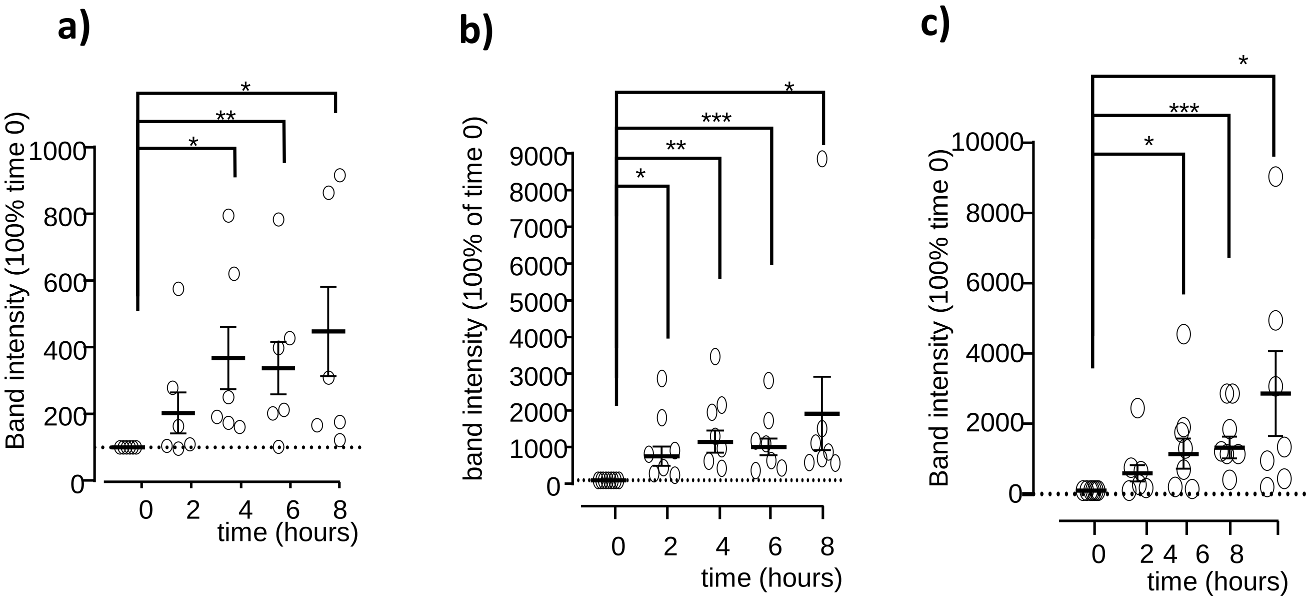

Supplementary Fig. 2 H_2S producing enzymes cellular remodeling in HL-1 cardiomyocytes via regular interval pacing. Summary mean (± s.e.m) band intensities of MPST **(a)** CBS **(b)** and CTH **(c)** as determined using ImageJ software at different pacing time periods (2- 8 hr) showing increased levels of CTH, CBS and MPST when compared to controls in unstimulated conditions (time 0). For all panels * P < 0.05, ** P < 0.01, *** P < 0.001 (n=6-7 experiments; unpaired t test).

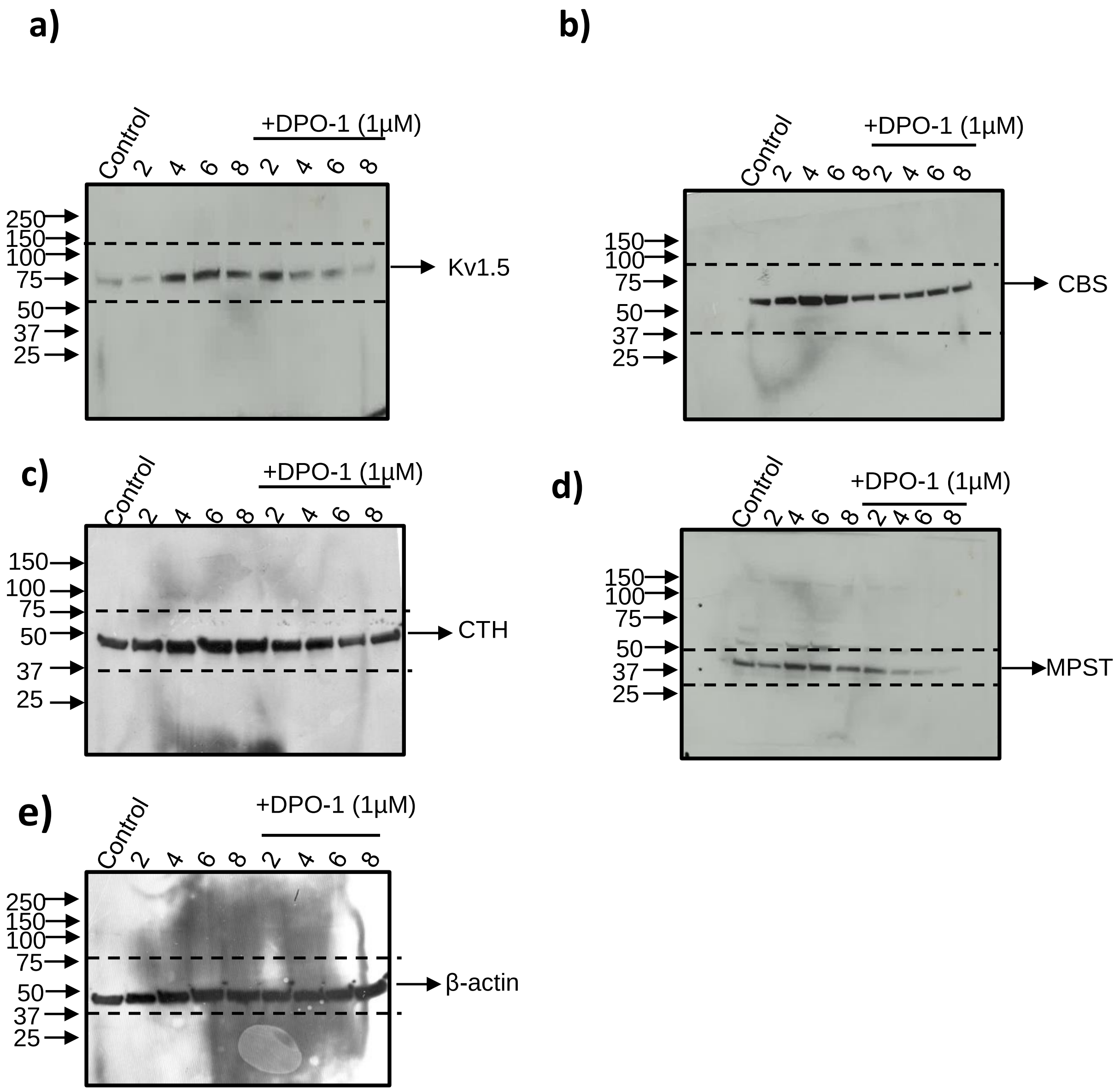

Supplementary Figure 3. **inhibition of Kv1.5 upregulation results in reduced cellular remodeling of H_2S-producing enzymes in HL-1 atrial cardiomyocytes.** Immunoblots scans showing Kv1.5 **(a)**, H_2S producing enzymes; CBS **(b)** CTH **(c)** MPST **(d)** following pacing. DPO-1, Kv1.5 inhibitor, was added to the media and for the experiment duration, HL-1 were subjected to interval pacing, paced cells without DPO-1 were used as controls. HL-1 cell lystae was obtained at 2, 4, 6, 8 hours stored at -20C until needed. Samples (30µg) were loaded onto SDS-PAGE, transferred onto PVDF membranes and processed as descried in methods. No corrections applied on the tone curve of the scanned images, images were resized to fit layout for the final used images. dashed lines used to show protein bands with the expected molecular weight for all blots. β-actin **(e)** was used as loading control.

a)

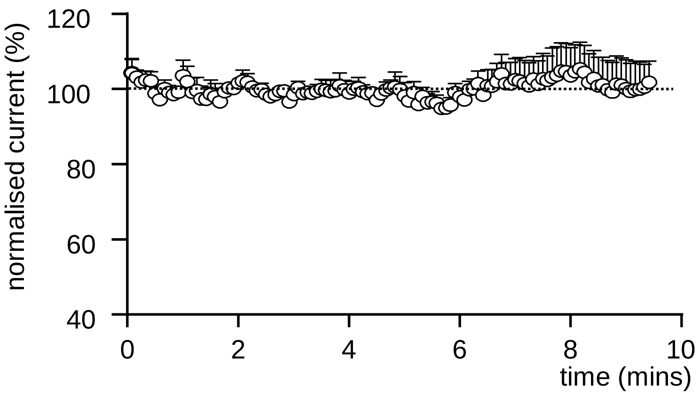

b)

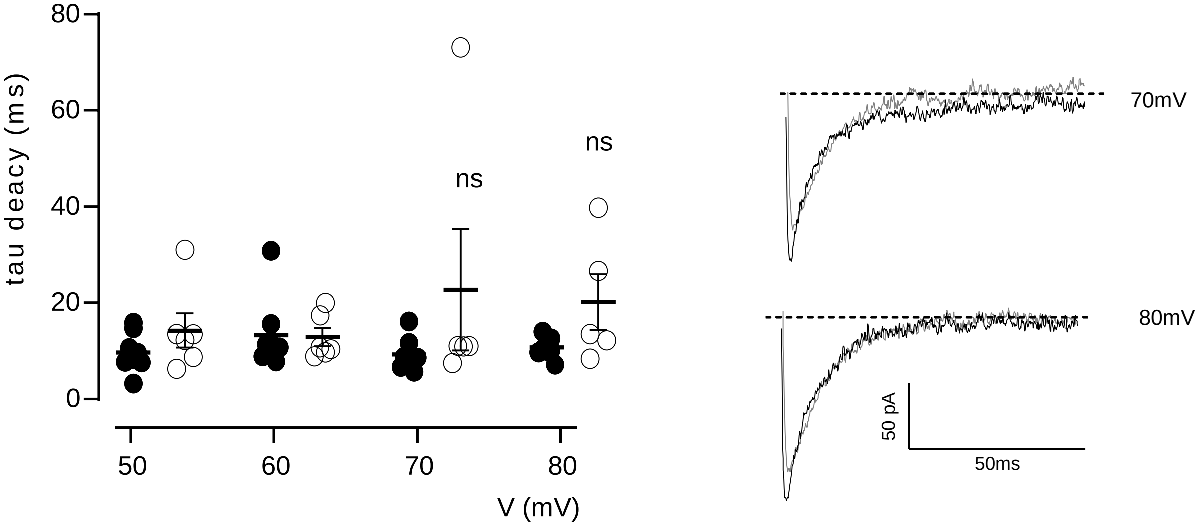

Supplementary Fig. 4 Recording of hKv1.5 in HEK293 cells (a) Mean (±s.e.m) time-series plot of control conditions illustrating normalized peak current amplitudes evoked by step depolarizations from -70mV to +50mV. In all of these experiments, the variation in normalized peak currents remained <5%, and small fluctuations persist throughout the duration of the experiments. The peak amplitudes during the control period had no drift and had coefficients of variation of <3.33% (n=8). **(b) s**howing the rate of recovery from inactivation obtained from tail current decay by fitting hKv1.5 Na^+ tail currents recorded in the recovery potential of −80mV in the in the absence (filled circles) and presence of H_2S-forming compound NaHS (open circles). Tail currents were fitted with single exponentials and time constants of Na^+ current inactivation versus the pre-pulse depolarization voltage presented as mean ±s.e.m (left; 5-8 cells for each point) for these examined potentials. Although it looks that there is an increase in rate of inactivation at the highest depolarizing potentials of +70mV and +80mv in the presence of H_2S, this was not statistically significant when compared to controls. The representative traces (right) for these higher test potentials from the same cell in the absence (black) and presence of H_2S-forming compound NaHS (grey).

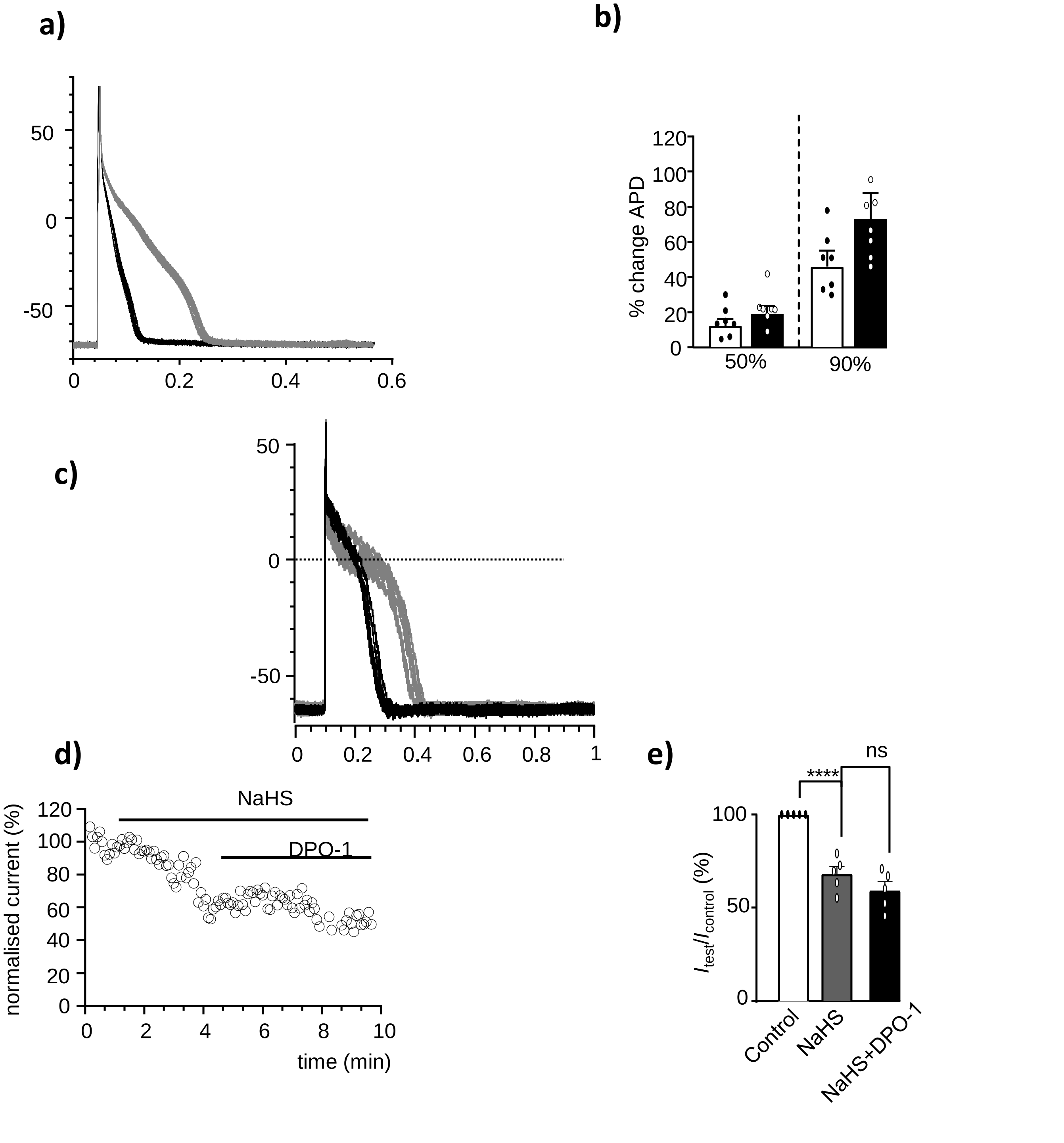

Supplementary Fig. 5 (a) Augments of action potentials recorded in rat atrial myocytes following DPO-1 perfusion, action potential variability before (black) and during (grey) perfusion of Kv1.5 inhibitor DPO-1 superimposed 20 APs in each condition. **(b)** Mean ± sem bar graph percentage of change in APD$_{90}$ calculated in rat atrial myocytes caused by DPO-1 (white,1 μM; n=7) or NaHS (black,100 μM; n=7). **(c)** showing a second example of AP variability in HL-1 before (black) and during (grey) perfusion of of H_2S-forming compound NaHS, superimposed 20 APs in each condition. **(d)** Example of time-series plot illustrating normalized peak current amplitudes recorded in HL-1 cell, H_2S was applied via H_2S-releasing compound NaHS followed by DPO-1. **(e)** Bar graph showing % normalized mean (± s.e.m; n=5) effect of NaHS and Kv1.5 inhibitor DPO-1 on outward K^+ currents peak amplitude measured at +50mV.

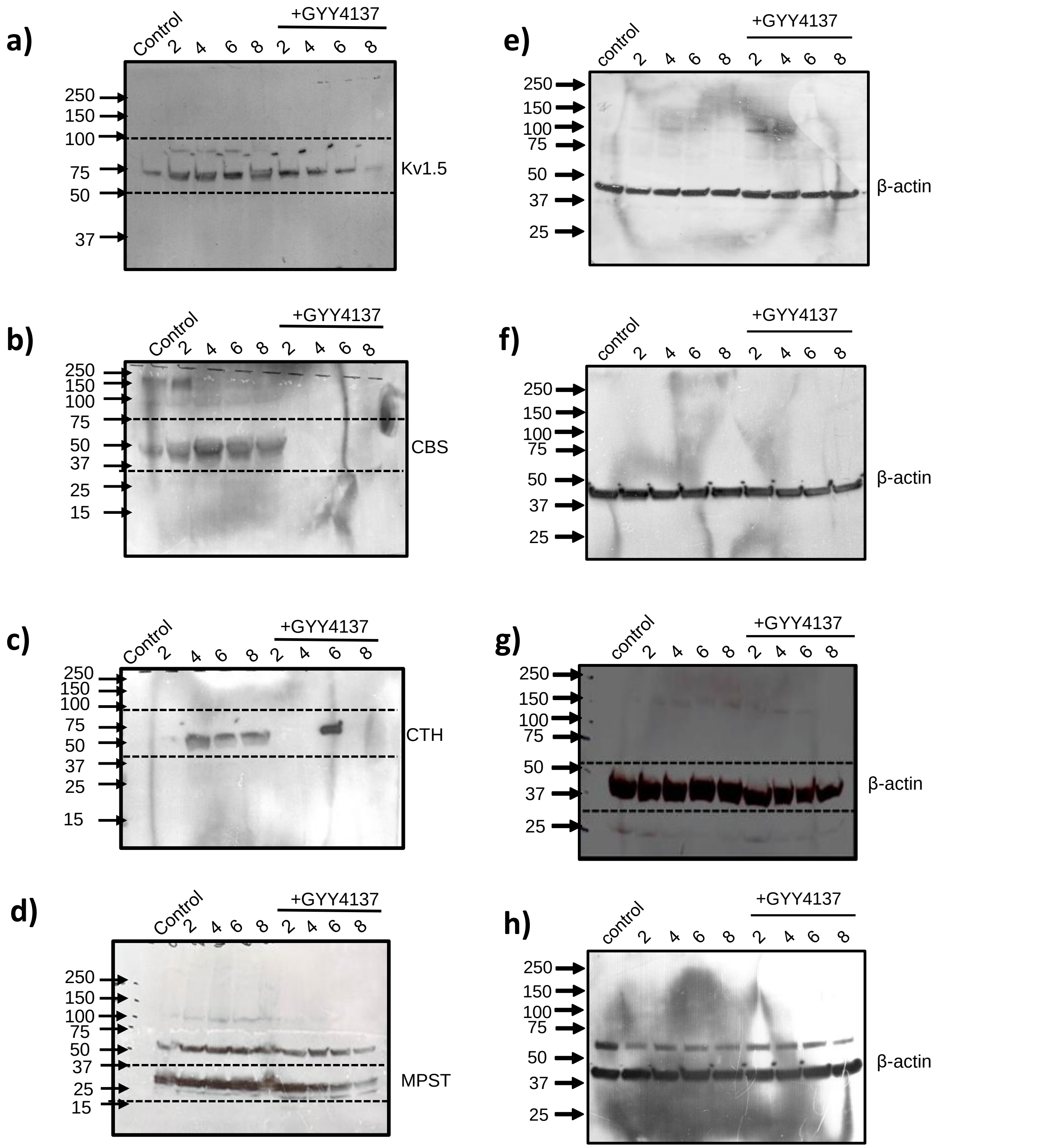

Supplementary Fig. 6. Full western blots corresponding to Fig 4. c, hydrogen sulfide reduced cellular remodeling in paced HL-1 atrial cardiomyocytes full blot scans with cropped lines as indicated by dashed lines. HL-1 Cell Lystae was obtained from paced cells at 2, 4, 6 and 8 hours time points either with or without supplementation of the slow-releasing hydrogen sulfide donor, GYY4137 (indicated with horizontal bar). **(a)** anti-Kv1.5 potassium channel, clone K7/45 (UC Davis/NIH NeuroMab Facility) with expected molecular weight of approximately 70 kDa band. **(b)** anti-cystathionine β-synthase antibody (CBS; Santa Cruz Biotechnology) with expected molecular weight of approximately 63 kDa band. **(c)** cystathionine γ-lyase antibody (CTH; Sigma-Aldrich) with expected molecular weight of approximately 45 kDa band. **(d)** anti-Mercaptopyruvate sulfurtransferase antibody (MPST; Sigma-Aldrich) with expected molecular weight of approximately 33 kDa band and **(e-h)** loading control β-actin (Sigma-Aldrich).

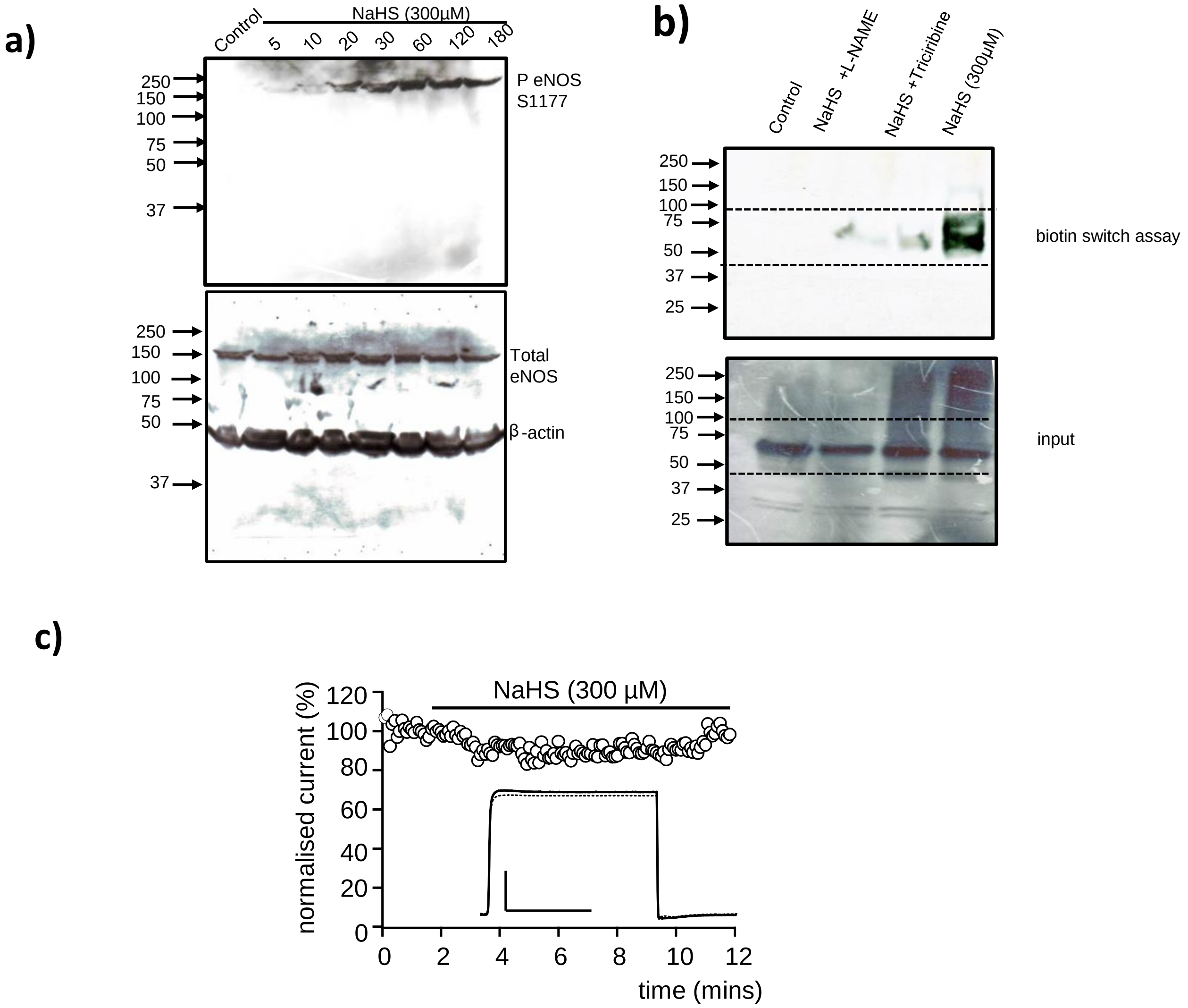

Supplementary Fig. 7. **Full western blots corresponding to Fig 5. c Inhibition of Kv1.5 by H_2S requires NO formation** full blot scans with cropped lines as indicated by dashed lines. **(a)** increased phosphorylation of eNOS at S1177, cell lysate was obtained from a HEK293 cell stably expressing hKv1.5 treated with H_2S releasing molecule NaHS (300uM) at time points shown on the heading of the blots and indicated with horizontal line, control represent cells not treated with H_2S, anti-Phospho-eNOS (Ser1177) antibody (Cell Signaling Technology) with expected molecular weight detection band of approximately140 kDa (*upper*) and anti-eNOS antibody (Cell Signaling Technology) with expected molecular weight detection band of approximately140 kDa (*lower*) and the loading control β-actin. **(b)** western blotting showing nitrosylation via the biotin-switch assay following HEK293 expressing hKv1.5 treatment with NaHS (300µM) alone or with NaHS (300µM) + L-NAME (1mM) or NaHS (300µM) + triciribine (5µM) to block NO formation, as indicated on image heading, control represent cells not treated with H_2S (*upper*) input level of hKv1.5 for each condition (*lower*). **(c)** a second time-series example plot showing the lack of effect of H_2S donor NaHS on normalized peak current amplitudes obtained from pre-treated HEK293 cell stably expressing human Kv1.5 (hKv1.5) with L-NAME. Inset shows example currents before and during NaHS exposure (vertical scale bar 1nA; horizontal scale bar = 50 ms)
