# Supplementary material for: Inhibition of the voltage-gated potassium channel Kv1.5 by hydrogen sulfide attenuates remodeling through S-nitrosylation-mediated signaling
Source: Commun Biol. 2023 Jun 19;6:651. doi: 10.1038/s42003-023-05016-5 (PMC10279668; doi:10.1038/s42003-023-05016-5)
Supplement: Supplementary file 4 — Reporting Summary [file 42003_2023_5016_MOESM4_ESM.pdf]

nature portfolio

Corresponding author(s): Moza Al Owais

Last updated by author(s): May 22, 2023

Reporting Summary

Nature Portfolio wishes to improve the reproducibility of the work that we publish. This form provides structure for consistency and transparency in reporting. For further information on Nature Portfolio policies, see our Editorial Policies and the Editorial Policy Checklist.

Statistics

For all statistical analyses, confirm that the following items are present in the figure legend, table legend, main text, or Methods section.

n/a	Confirmed	
☐	☒	The exact sample size (*n*) for each experimental group/condition, given as a discrete number and unit of measurement
☐	☒	A statement on whether measurements were taken from distinct samples or whether the same sample was measured repeatedly
☐	☒	The statistical test(s) used AND whether they are one- or two-sided<br>*Only common tests should be described solely by name; describe more complex techniques in the Methods section.*
☒	☐	A description of all covariates tested
☐	☒	A description of any assumptions or corrections, such as tests of normality and adjustment for multiple comparisons
☐	☒	A full description of the statistical parameters including central tendency (e.g. means) or other basic estimates (e.g. regression coefficient) AND variation (e.g. standard deviation) or associated estimates of uncertainty (e.g. confidence intervals)
☐	☒	For null hypothesis testing, the test statistic (e.g. *F*, *t*, *r*) with confidence intervals, effect sizes, degrees of freedom and *P* value noted<br>*Give P values as exact values whenever suitable.*
☒	☐	For Bayesian analysis, information on the choice of priors and Markov chain Monte Carlo settings
☒	☐	For hierarchical and complex designs, identification of the appropriate level for tests and full reporting of outcomes
☒	☐	Estimates of effect sizes (e.g. Cohen's *d*, Pearson's *r*), indicating how they were calculated

Our web collection on statistics for biologists contains articles on many of the points above.

Software and code

Policy information about availability of computer code

Data collection	Patch Clamp data was collected using pCLAMP 10.
Data analysis	Data analyzed using Excel (Microsoft, UK) and Origin (Northampton, MA) software.<br>Band intensities were measured using ImagJ analysis software.

For manuscripts utilizing custom algorithms or software that are central to the research but not yet described in published literature, software must be made available to editors and reviewers. We strongly encourage code deposition in a community repository (e.g. GitHub). See the Nature Portfolio guidelines for submitting code & software for further information.

Data

Policy information about availability of data

All manuscripts must include a data availability statement. This statement should provide the following information, where applicable:

- Accession codes, unique identifiers, or web links for publicly available datasets
- A description of any restrictions on data availability
- For clinical datasets or third party data, please ensure that the statement adheres to our policy

The original contributions presented in the article/supplementary material are included in this study, any further inquiries can be directed to the corresponding author.

Human research participants

Policy information about studies involving human research participants and Sex and Gender in Research.

Reporting on sex and gender	NA
Population characteristics	NA
Recruitment	NA
Ethics oversight	NA

Note that full information on the approval of the study protocol must also be provided in the manuscript.

Field-specific reporting

Please select the one below that is the best fit for your research. If you are not sure, read the appropriate sections before making your selection.

☒ Life sciences ☐ Behavioural & social sciences ☐ Ecological, evolutionary & environmental sciences

For a reference copy of the document with all sections, see nature.com/documents/nr-reporting-summary-flat.pdf

Life sciences study design

All studies must disclose on these points even when the disclosure is negative.

Sample size	Based on our experience for electrophysiology experiments (voltage and current clamp) a minimum of 6-8 repeats is required for data to obtain statistical validity of observations.<br>Repeats of 3-7 were achieved for all cell culture experiments used for immunoblots and band intensities statistical analysis.<br>Atria tissue from 5 hearts were used for this study.<br>Statistical comparisons with $p < 0.05$ taken as statistically significant
Data exclusions	Data excluded if;<br>1) Baseline of cells did not remain stable during experiment, or if cell developed a leak throughout the experiment.<br>2) Significant increase in series resistance occurred (>20%), then experiment was eliminated.<br>3) A technical failure occurs during experiment.
Replication	Experiments were replicated successfully.
Randomization	N/A
Blinding	N/A

Reporting for specific materials, systems and methods

We require information from authors about some types of materials, experimental systems and methods used in many studies. Here, indicate whether each material, system or method listed is relevant to your study. If you are not sure if a list item applies to your research, read the appropriate section before selecting a response.

Materials & experimental systems

n/a	Involved in the study	
☐	☒	Antibodies
☐	☒	Eukaryotic cell lines
☒	☐	Palaeontology and archaeology
☐	☒	Animals and other organisms
☒	☐	Clinical data
☒	☐	Dual use research of concern

Methods

n/a	Involved in the study	
☒	☐	ChIP-seq
☒	☐	Flow cytometry
☒	☐	MRI-based neuroimaging

Antibodies

Antibodies used	The following antibodies were used for detection;<br>Anti-Kv1.5 potassium channel, clone K7/45 (UC Davis/NIH NeuroMab Facility)<br>Cystathionine β-synthase antibody (CBS; Santa Cruz Biotechnology)<br>Cystathionine γ-lyase antibody (CTH; Sigma-Aldrich)<br>Mercaptopyruvate sulfurtransferase antibody (MPST; Sigma-Aldrich)<br>β-actin (Sigma-Aldrich).<br>Anti-Phospho-eNOS (Ser1177) antibody (Cell Signaling Technology)<br>Anti-eNOS antibody (Cell Signaling Technology)
Validation	Validation documents of the antibodies were provided by the manufacturers.<br>https://neuromab.ucdavis.edu/datasheet/K7_45.pdf<br>https://www.scbt.com/p/cbs-antibody-b-4<br>https://www.sigmaaldrich.com/GB/en/specification-sheet/SIGMA/HPA023300<br>https://www.sigmaaldrich.com/GB/en/specification-sheet/SIGMA/HPA001240<br>https://www.sigmaaldrich.com/GB/en/substance/monoclonalantibactinantibodyproducedinmouse1234598765<br>https://www.cellsignal.com/products/primary-antibodies/phospho-enos-ser1177-antibody/9571<br>https://www.cellsignal.com/products/primary-antibodies/enos-antibody/9572

Eukaryotic cell lines

Policy information about cell lines and Sex and Gender in Research

Cell line source(s)	HL-1 (Sigma-Aldrich)<br>HEK293 (ATCC)
Authentication	Validation documents were provided by the manufacturers.<br>https://www.sigmaaldrich.com/GB/en/product/mm/scc065?<br>gclid=EAIaIQobChMIvri7jsTE_glVwcvVCh350QB9EAAYAiAAEgJvFPD_BwE&gclsrc=aw.ds<br>https://www.atcc.org/products/crl-1573
Mycoplasma contamination	Cell lines tested negative for mycoplasm contamination.
Commonly misidentified lines<br>(See ICLAC register)	*Name any commonly misidentified cell lines used in the study and provide a rationale for their use.*

Animals and other research organisms

Policy information about studies involving animals; ARRIVE guidelines recommended for reporting animal research, and Sex and Gender in Research

Laboratory animals	Wistar rats (150-200g)
Wild animals	N/A
Reporting on sex	N/A
Field-collected samples	N/A
Ethics oversight	animals were humanely euthanized in accordance with UK Home Office Guidance on the 331 Operation of Animals (Scientific Procedures) Act 1986 and Institutional guidelines.

Note that full information on the approval of the study protocol must also be provided in the manuscript.
